# Perceptions of Neurosurgery among Medical Students and Interns: A National Cross-Sectional Study

**DOI:** 10.3390/medicina58081120

**Published:** 2022-08-18

**Authors:** Ibrahim Alnaami, Mohammad Tauheed Ahmad, Mohammed Abid Khan, Khaled A. Amer, Abdullah Alsaedan, Abdulaziz Alanazi, Sarah Alkhonizy, Abdulaziz Alamri

**Affiliations:** 1 Division of Neurosurgery, Department of Surgery, College of Medicine, King Khalid University, Abha 62529, Saudi Arabia; 2 Department of Medical Education, College of Medicine, King Khalid University, Abha 62529, Saudi Arabia; 3 College of Medicine, Shaqra University, Shaqra 11961, Saudi Arabia; 4 College of Medicine, Alqassim University, Alqassim 52571, Saudi Arabia; 5 College of Medicine, Alfaisal University, Riyadh 11533, Saudi Arabia; 6 Department of Surgery, College of Medicine, King Khalid University, Abha 62529, Saudi Arabia

**Keywords:** specialization perceptions, neurosurgery, medical students, interns, medical education

## Abstract

**Background:** Neurosurgery is considered one of the most admired surgical specializations. Still, as evidenced by observations over the years, it is not amongst the top choices of medical students in Saudi Arabia. This study will help in understanding the perception and attitudes of medical students and interns toward the neurosurgery specialization, which will aid in developing strategies to increase the interest of undergraduates in neurosurgery specialization and bridge the human resource gap in this vital specialty. **Objectives:** This study aimed to assess perceptions, attitudes, and gender differences of medical students and interns toward neurosurgery. The influence of demographic factors on perceptions of neurosurgery of interns and senior medical students in Saudi Arabia was also investigated. **Methodology:** We conducted a cross-sectional study on a convenience sample of medical students (clinical years) and interns studying in different colleges of medicine in Saudi Arabia. A total of 1014 responses were included in this study (518 males and 496 females). The questionnaire comprised of two parts: the first part carried general descriptive questions, while the second part had 24-item Likert scale-based questions. **Results:** Around 40% of medical students and 26% of interns agreed or strongly agreed to the statement ‘I would consider a career in neurosurgery. Around 70% of both interns and students agreed or strongly agreed with the statement ‘Huge prestige is attached to neurosurgery’. The responses to the statement ‘Neurosurgery is a male specialty’ was significantly different between genders, as 36.5% of the male respondents agreed or strongly agreed with the statement while just 12.5% of the females responded likewise (*p* = 0.000). Only 23% of participants reported having completed a rotation in neurosurgery. About 44.7% of respondents were interested in taking an elective rotation in neurosurgery, while the remaining respondents (55.3%) responded with a negative answer. Only 23% of participants had completed an undergraduate rotation in neurosurgery. More males (65.3%) agreed or strongly agreed with the statement ‘Work as a neurosurgery specialist can impede family life as compared to females (57.5%), which was highly significant (*p* = 0.000). More medical students (45.1%) responded in the agreement or strong agreement in response to the statement’ Neurosurgery should be a compulsory rotation during internship’ as compared to interns (25.8%). Around 65% of the respondents agreed or strongly agreed that teachers/seniors have a great influence on students’ specialization choices. Around 67% of the respondents foresaw a purely clinical role for themselves after graduation, while 17% were interested in a clinical–academician role. **Conclusions:** The study reflected a generally positive attitude towards neurosurgery amongst medical students and interns. Interest in neurosurgery as a specialization can be strengthened by enhancing the exposure of students and interns to the specialty. Teachers and seniors can be useful influencers to motivate students to consider neurosurgery specialization.

## 1. Introduction

Neurosurgery is an exciting surgical specialty that requires precise skills and utilizes cutting-edge technology to perform intricate surgeries, most of the time on critical patients. Over the past two decades, there has been a difference in the findings regarding the popularity of neurosurgery as a specialization of choice amongst medical students [1,2]. A number of reasons have been cited for the lack of interest in neurosurgery viz. lack of adequate exposure to neurosurgery during their undergraduate years, neurosurgery requiring a higher degree of skills that are challenging to acquire, as well as the longer duration of neurosurgery residency programs [3]. In data from 2011, despite a greater number of medicine graduates passing out of medical colleges in Canada, there was no change in the number of students applying for the neurosurgery residency programs. The cause behind this is stable.

The number was explained as reduced exposure to neurosurgery in the new curriculum [4]. In the USA, there was a decline in neurosurgery residency applications as lifestyle and work hours significantly influenced the decisions of medical students’ residency choices [5]. In China, the perception of medical students toward neuroscience and neurosurgery has been reported as unfavorable, due to which they have lower levels of knowledge of neuroscience topics [6]. From 2015 to 2017, neurosurgery has been consistently ranked in the top five choices amongst the pass outs seeking surgical training positions (year 1) in the United Kingdom [7]. A study carried out at Tabuk University, Saudi Arabia, reported that neurosurgery is the most difficult specialty amongst medical students as regards their specialization choices [8]. In Riyadh, Saudi Arabia, a study shed light on the attitudes and perceptions of neurosurgery as a specialty and to aid the process of assimilating neurosurgery knowledge. It found that almost 85% of the students are unwilling to opt for neurosurgery as a specialization, mostly citing work–life balance issues [9]. Similar findings regarding the issue of work–life balance being a major concern to be considered were reported from another study in Canada [4]. To increase students’ interest in neurosurgery, they should be exposed to neurosurgery clinics as early as possible to orient them to new advancements and should be encouraged to read more about the neurosciences [10].

Significant technological advancements are taking place continuously in the field of medicine. Multiple factors could have unfavorable effects on the interns and medical students in Oman toward choosing neurosurgery as a specialty [11]. Another recent study listed some factors affecting the specialization choices of medical students with respect to neurosurgery and concluded that undergraduate preparations, research experience, and competitiveness may impact the specialization choice [12]. Another fact to consider is historical under representation of women in surgical specialties. A study conducted in the USA reported potential associations between gender and specialization choices with respect to neurosurgery [13].

In a survey of undergraduate medical students conducted at King Saud University in 2011, while general surgery was opted for by 27.4% of the respondents and ENT-Ophthalmology was opted for by 24.6% of respondents, only 15.7% of the respondents had a preference for neurosurgery [14].

In another study of medical students and interns conducted in Saudi Arabia in 2013, only 13 out of 379 medical students surveyed reported an interest in specializing in neurosurgery, while 56 students preferred internal medicine, 34 preferred general surgery, and 27 participants each chose pediatrics and emergency medicine [15].

According to an estimate, only 17.7% of the doctors working in neurosurgery specialty in Saudi Arabia were Saudi Arabian nationals, which is one of the lowest for any specialization. This ratio is lower than that for gastroenterology, general surgery, oncology, and many other specializations. Additionally, given the fact that neurosurgeons are required for the management of emergency trauma cases, and their availability can be crucial even in remote hospitals, this shortage of neurosurgeons is a matter of concern [15]. The last census from the Saudi Association of Neurological Surgery announced their 2022 annual meeting, mentioning only around 400 male and female neurosurgeons in all health sections, Ministry of Health, University hospitals, military hospitals, National Guard hospitals, and Private hospitals included. All neurosurgery pathologies—cranial as well as spine—are dealt with in super specialty hospitals, which are present in all regions and most of the major cities of Saudi Arabia. However, the complexity of the surgeries performed differs as per the level of the hospital being secondary, tertiary, or quaternary.

The neurosurgical training program in Saudi Arabia consists of two parts—junior and senior training—summing up to a total of 6 years of training. There are strict guidelines for selection, training as well as certification. The Saudi Commission for Health Specialties (SCFHS) is the professional body responsible for the registration and regulation of all healthcare specialists, including neurosurgeons. With the setting up of multistate-of-the-art tertiary care centers in the Kingdom of Saudi Arabia in the past decades, the opportunities for neurosurgeons to perform more complex surgeries have increased.

The students usually join the College of Medicine between the ages of 17 and 18 years and graduate at the age of 24–25 years. Medical students start their clinical years (year 4–year 6) at the age of 20–21 years. Students who participated in the questionnaire were between the ages of 20 and 24, whereas the ages of the interns were between 24 and 25 years.

The availability of qualified neurosurgeons at a greater number of hospitals, especially in remote areas, will improve the outcomes of patients requiring urgent surgical interventions and also ensure the growth of the specialty. It is pertinent that steps are undertaken to attract students toward the neurosurgery specialization. This study is the first national-level multicentric study in Saudi Arabia to understand the experiences, perceptions, and attitudes towards neurosurgery. The influence of demographic factors including gender, past experience and interest in specialty, and stereotypes on perceptions of neurosurgery in Saudi Arabia was also investigated. This study will help in guiding efforts to popularize neurosurgery amongst upcoming medicine graduates and will hopefully help in filling this human resource health gap.

## 2. Methods and Study Design

### 2.1. Methodology

This study was carried out in the Kingdom of Saudi Arabia from March 2021 to July 2021. This cross-sectional study included 1014 students (518 males and 496 females) who participated from medical colleges across the Kingdom of Saudi Arabia. Multiple strategies were employed to reach out to the target population viz online distribution through social media, emails sent to university student email lists, face-to-face approach for increasing the response rate as far as possible as well as student mobilization through student clubs at medical colleges. The estimated response rate achieved was around 20–25 percent.

The IRB was obtained from the IRB board of King College University. After signing the consent, participants recorded their responses based on a structured and pre-tested questionnaire. Entries in the form of incomplete questionnaires were excluded from the study.

In Saudi Arabia, the majority of medical colleges use the British system of study in medical colleges, where seven years are required for an undergraduate degree. The first three years are basic sciences, whereas the 4th, 5th, and 6th years are the clinical years. The 7th year is an internship where the intern is expected to spend 12 months in clinical rotations at accredited hospitals inside or outside Saudi Arabia.

The questionnaire was designed based on previous studies [1]. This questionnaire is comprised of two sections, as listed in Table 1. The first section included demographic information (gender, year of study, Nationality). The second section included different factors such as past experience and interest in specialty, stereotypes, and personnel factors that might influence perceptions and attitudes of medical students and interns toward neurosurgery. Twenty-four items were mentioned in the survey and scored, following a Likert ranking scale 1: strongly agree, 2: agree, 3: not sure, 4: disagree, and 5: strongly disagree) as it is illustrated in Table 1. Few questions were added to the questionnaire from another study [1] after validation by three professors from the department of surgery at the Medical College of King College University, and then we validated the questionnaire by eliciting responses and feedback from 15 randomly selected medical students in the final years to rule out any comprehension issues.

### 2.2. Data Analysis

The data thus collected were analyzed using SPSS version 16.0. Percentages were calculated for the demographic variables, and mean values along with standard deviation were calculated for the Likert scale questions. Inter-group variability was estimated using *p* Values. Independent groups *t*-test was also employed to perform multiple comparisons of intra-group variability. A *p*-value of less than 0.05 was considered statistically significant.

## 3. Results

### 3.1. Demographic Information and Distribution of Respondents to Question Items

**Figure 1:**
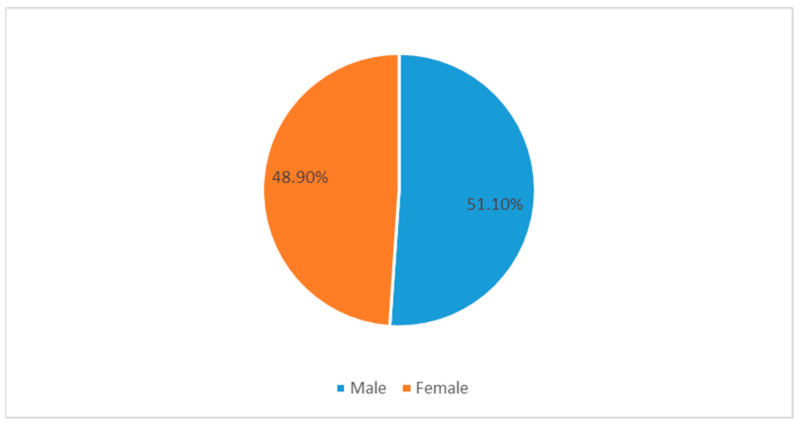
Pie chart for the gender distribution of respondents.

**Figure 2:**
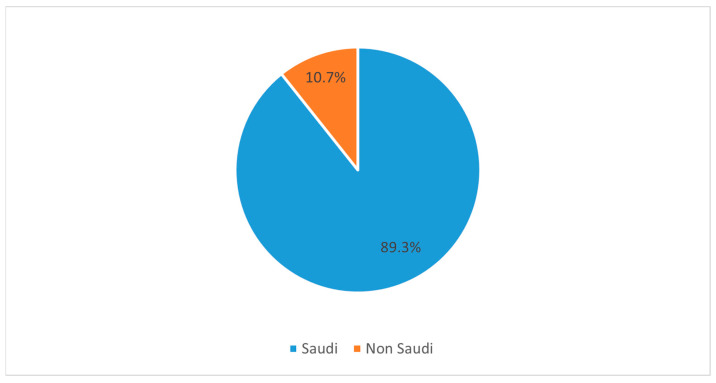
Pie chart illustrates the distribution of participants based on their nationality.

**Figure 3:**
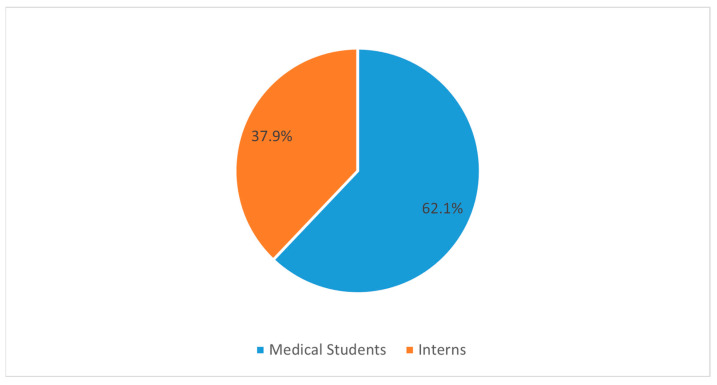
Pie chart demonstrates the distribution the respondents based on their seniority being medical students versus interns.

A total of 1014 responses were recorded from study participants from medical colleges across the Kingdom of Saudi Arabia. The gender-wise, nationality-wise, and seniority-wise distribution is illustrated in Figure 1, Figure 2 and Figure 3, respectively. Government medical colleges in Saudi Arabia generally admit Saudi nationals in the Bachelor of Medicine, Bachelor of Surgery (MBBS) program. However, there are a number of private medical colleges which admit students of other nationalities. Among the total number of participants filling the questionnaire, there were 518 males (51.01%) and 496 females (48.90%), as listed in Figure 1.

In our survey, 10.7% of the responses were from non-Saudi nationals, whereas most respondents were from Saudi Arabia, with a distribution percentage of 89.3% (Figure 2). In our study, we included only students who had already entered the clinical years as well as interns. Interns, in general, have greater exposure to clinical settings, especially in the emergency department, where there is a greater chance of exposure and opportunity for care of neurosurgery patients, for example, trauma patients. Hence, our study divided the respondents into two broad groups into these lines for comparison viz. medical students and interns. Our respondents comprised a distribution percentage of 62.1% for medical students and 37.9% for interns (Figure 3).

**Figure 4:**
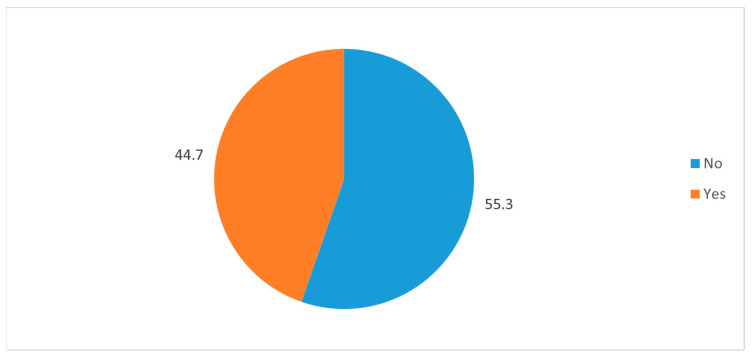
Pir chart reveals the distribution the respondents based on their response to the statement “would you be interested in taking an elective rotation in neurosurgery to get more exposure of the neurosurgery specialty?”.

**Figure 5:**
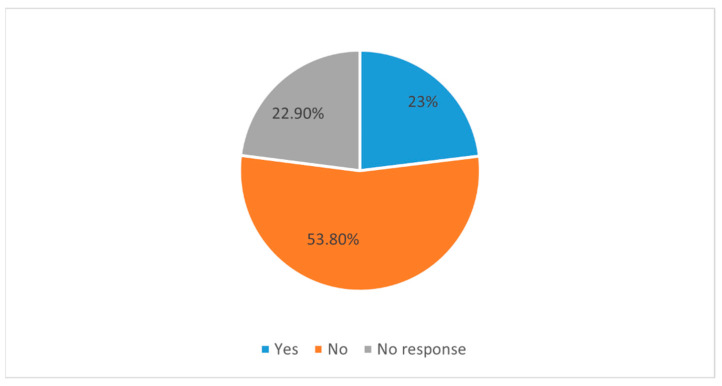
Pie chart shows distribution of respondents based on their self-reported completion of the undergraduate neurosurgery rotation.

**Figure 6:**
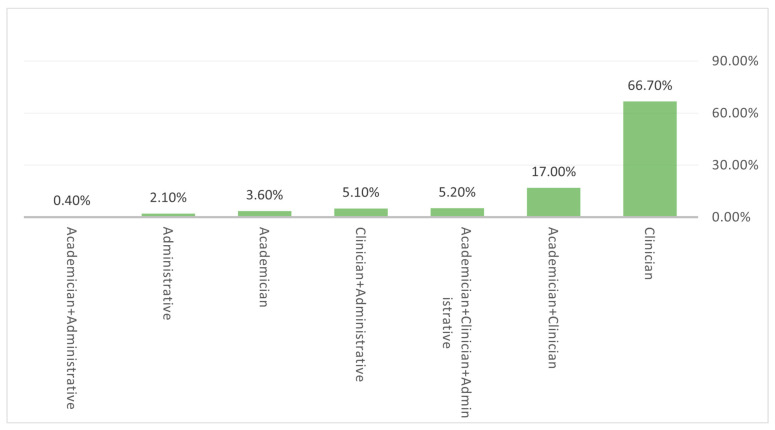
Bar chart illustrates the distribution of respondents based on their response to the question “what kind of role do you see for yourself in your future career?”.

In order to gauge their general interest in neurosurgery specialty, the study subjects were asked if they would be interested in taking an elective rotation in neurosurgery. Less than half of the respondents (44.7%) responded in the affirmative, while the rest (55.3%) responded with a negative answer (Figure 4). Exposure to the discipline of neurosurgery was assessed by asking the respondents if they had completed an undergraduate rotation in neurosurgery. More than half (53.8%) of the respondents replied in the negative to this question, while only 23% of respondents had completed an undergraduate rotation in neurosurgery (Figure 5). In response to the question regarding the perception of their future career roles, almost 67% of the respondents saw themselves going into a purely clinical role, while 17% of the respondents had reportedly envisioned clinician–academician roles for themselves, and 3.60% of respondents had reported academicians (Figure 6).

**Figure 7:**
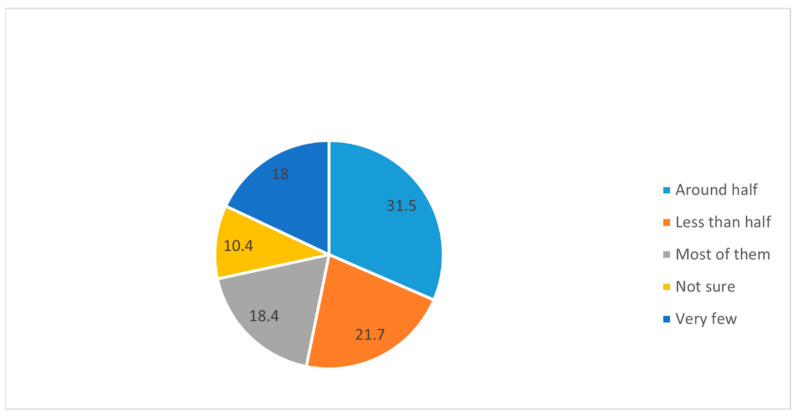
Pie chart shows the distribution of subjects based on their responses to the question “what proportion of classmates/college-friends whom you know well have made their minds regarding their specialization choices?”.

**Figure 8:**
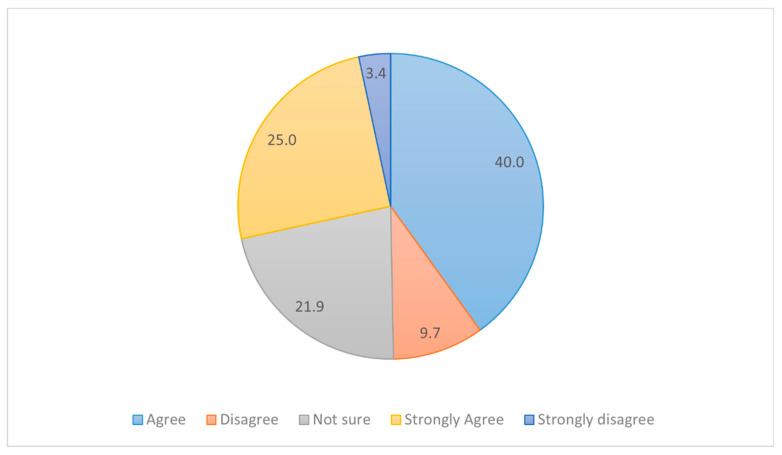
Pie chart reveals the distribution of subjects based on their response to the statement “college teachers/seniors have a great influence on any student’s specialization choice after MBBS”.

In order to get an idea of the future specialization-related thoughts of the study group, we asked them about their perception of other students’ decisions regarding the specialization choices. Around half of the respondents believed that around half (31.5%) to most of them (students/interns) have made up their minds regarding their specialization choices (Figure 7). Around 65% of the respondents ‘agree’ and ‘strongly agree’ that teachers/seniors have a great influence on the students’ specialization choice after MBBS (Figure 8). Additionally, 40%, 25%, 9.7%, and 3.4% of participants respectively responded agree, strongly agree, disagree, and strongly disagree in regard to the perception of the great influence of seniors on specialization choice after MBBS (Figure 8).

### 3.2. Factors Affecting Perceptions and Attitudes of Neurosurgery

Concerning differences in perceptions and choices regarding specialization across the genders in Saudi Arabia, our study compared the mean Likert scales for male and female respondents (Table 2). The mean Likert scales were compared amongst these two groups of respondents (medical students and interns), as listed in Table 3. Along with the mean Likert scale measures, standard deviation as well as the mean Likert scale responses for each of the two groups along with standard deviation and *p*-value were calculated for each of the statements. In Table S1 (Supplementary), the two comparisons between the genders and between medical students and interns are summarized based on the percentages of those who strongly agreed or agreed with the given statements. When compared across genders, a significant difference was found between the male and female respondents as regards to entry competitiveness perception (*p* = 0.004), perception of neurosurgeon’s intelligence (*p* = 0.000), as well as perception about neurosurgery being a ‘depressing specialty’ (*p* = 0.003), ‘interesting specialty’ (*p* = 0.000) or a ‘male specialty’ (*p* = 0.000) or ‘ neurosurgery impeding family life’(*P* = 0.000) or ‘taking an elective rotation in neurosurgery’ (*p* = 0.020) or ‘important neurosurgery’ (*p* = 0.030) or ‘long operating hours required for neurosurgery’ (*p* = 0.019) or ‘a compulsory neurosurgery rotation in internship’ (*p* = 0.014) or ‘completing emergency department rotation for interns’ (*p* = 0.003) or ‘an interesting neurological illness ’ (*p* = 0.000) (Table 2).

The gender difference in the responses to the statement that ‘neurosurgery is a male specialty’ was remarkable, as 36.5% of the male respondents agreed or strongly agreed to the statement, while just 12.5% of the females responded likewise (Table 4). There was a generally positive attitude toward the neurosurgery specialization among females. The males (43.6%) responded more in the agreement or strong agreement than females (37.7%) to the statement regarding neurosurgery being a depressing specialty (Table 4). To the statement that neurosurgery specialty can impede family life, more males (65.3%) agreed or strongly agreed with the statement as compared to females (57.5%), which shows a more favorable attitude to neurosurgery amongst females. Additionally, more females reported agreement or strong agreement to statements eliciting their responses if neurosurgery is an interesting specialty (Table 4). Moreover, females reported a strong agreement or agreement than males on many statements, including competitive training programs in neurosurgery (females: 55.5%, males: 49.4%), intelligent neurosurgeons (F: 76.6%, M: 67.4%), interesting neurosurgery as a specialty (F: 82.7%, M: 72%), long operating hours for neurosurgery (F: 89.1%, M: 87.6%), neurosurgical illness is challenging and interesting (F: 87.1%, M: 79.2%), and a compulsory neurosurgery rotation (F: 40.1% and M: 35.5%), as illustrated in Table 4.

From Table 3, a significant difference was found between medical students and intern respondents with regards to a career in neurosurgery (*p* = 0.000), completing undergraduate neurosurgery rotation for MBBS students (*p* = 0.000), completing emergency department rotation for interns (*p* = 0.001), requiring excellent manual dexterity for neurosurgeons (*p* = 0.027), being neurosurgery as an interesting specialty (*p* = 0.005), a compulsory neurosurgery rotation for internship (*p* = 0.000), and taking an elective rotation in neurosurgery specialty (*p* = 0.000). Comparing the medical students with interns, 40.2% of medical students and 26.6% of interns agreed or strongly agreed with the statement whether they would consider a career in neurosurgery (*p* = 0.000), as illustrated in Table S1 (Supplementary). Moreover, medical students agreed or strongly agreed more than interns with significant differences in many statements, including intelligent neurosurgeons (medical students: 74.1%, interns: 68%), requiring excellent manual dexterity for neurosurgeons (medical students: 83.7%, interns: 78.6%), neurosurgery as an interesting specialty (medical students: 78.1%, interns: 75.8%), impeding family life (medical students: 60.5%; interns: 63%) and being the neurosurgery rotation as a compulsory one in the internship (medical students: 45.1%, interns: 25.8%), as shown in Table S1 (Supplementary). In general, interns had a more negative attitude toward neurosurgery as compared to the medical students. Moreover, around 65% of both students and interns agreed or strongly agreed with the statement that seniors/teachers have a great influence on the student’s choice of specialization after MBBS. Table S1 (Supplementary). Further results comparing the differences in perception of neurosurgery between students who have been exposed to neurosurgery vs. students who have not been exposed to neurosurgery, as well as interns, are presented in (supplementary section Tables S2 and S3).

## 4. Discussion

In recent years, significant changes have taken place in the field of medicine, including breakthroughs in technology, research, and the entry of women students into the field. Interns and medical students’ perceptions of certain specialties can be influenced by a variety of circumstances; the majority of medical students dislike neurosurgery as a specialty [11]. However, according to our survey, nearly half of the respondents are interested in taking an elective rotation in neurosurgery. Additionally, in our survey, it was found that only 23% of participants had completed an undergraduate rotation in neurosurgery, which reflects the variation amongst colleges in Saudi Arabia regarding exposure to neurosurgery. In addition, it raises the question of whether the lack of such exposure has an impact on students’ perception of neurosurgery. According to our study, around 54% of medical students think that neurosurgery is a challenging and interesting specialty that could nourish and satisfy their ambitions. Other studies conducted in the past have come out with similar results regarding the importance of neurosurgery as a specialization [1]. Nevertheless, high prestige and income is a major factor behind the choice of neurosurgery as a specialization [9].

In our study, a significant difference was found between male and female respondents concerning the following statements: entry competitiveness perception, perception of neurosurgeon’s intelligence, as well as perception about neurosurgery being a depressing specialty, interesting specialty or a male specialty or neurosurgery impending family life or taking an elective rotation in neurosurgery or important neurosurgery or long operating hours required for neurosurgery or a compulsory neurosurgery rotation in an internship or completing emergency department rotation for interns or an interesting neurological illness. Moreover, our study revealed that the responses to the statement that ‘neurosurgery is a male specialty’ was remarkable, as 36.5% of the male respondents agreed or strongly agreed with the statement, while just 12.5% of the females responded likewise. Similarly, neurosurgery is still a male-dominated specialty, although the proportion of female medical graduates has been on the rise [1]. However, both male and female surgeons reported surgical careers to be satisfying [16]. Another study revealed that surgery was a masculine specialty and the deterrents for females in surgical careers still exist [17]. The number of women in neurosurgery is a topic that is always discussed at conferences, workshops and for sure, it has its own impact on the perception of neurosurgery; in the United States, for instance, women represented just 15% of incoming new neurosurgery residents, and few females had positions in upper academic echelons [18]. This variation is reflected significantly in leadership positions in the USA, which appear to be dominated by males [13]. Another study demonstrated that an overwhelming majority of resident and research grantees were males, with a male-to-female ratio of around 8:1 [12]. To make it worse, the female retention rate was also lower than that of males (83% vs. 94.7%), and female attrition was three times as frequent as male attrition (F: 17%, M: 5.3%) [19]. The identified reasons why women choose or leave surgical fields included perceived unequal treatment based on gender, lack of female role models/mentors, disparate salaries, home responsibilities, etc. [20,21]. According to a recent report by the Ministry of Health cited in the literature on the topic of women neurosurgeons in the Middle Eastern countries [22], there were only 10 women neurosurgeons in Saudi Arabia. Likewise, according to a 2019 report by the Saudi Commission for Health Specialties (SCFHS), there were nine active women consultant neurosurgeons, while there were 42 women residents in neurosurgery specialization. Women neurosurgeons have been active in Saudi Arabia for the last 25 years, and a few of them have distinguished themselves regionally and internationally. Such role models may inspire more women doctors to opt for neurosurgery specialization. In other studies on specialization choices of MBBS students conducted in Saudi Arabia, it has been found that surgery, in general, was the top choice for specialization amongst female medical students as well [23,24]. Hence, there is ample scope for the promotion of neurosurgery as a specialization amongst female medical students in Saudi Arabia.

Another aspect that showed similar responses amongst both males and females was the perception that neurosurgery is a depressing specialty, with a higher degree of agreement among male students. Moreover, with regard to the statement that the neurosurgery profession could impede family life, both males and females, as well as students and interns, are in agreement with that. This perception is noticed in many studies due to the seriousness of the pathologies that neurosurgeons deal with and the nature of the critical patients admitted to the service [11,25]. In a study on gender differences in perceptions and attitudes towards neurosurgery conducted in Germany recently [26], it was found that work–life balance was the single most important factor deterring students from taking up a career in neurosurgery. This was similar to the findings of our study. However, in the same study, male students were found to have a more positive attitude toward neurosurgery as compared to our study.

In a study conducted in Ireland, more than 90% of respondents agreed that neurosurgery necessitates long operating hours and a long training period, and 88% of respondents reported that neurosurgical training is prolonged. Of respondents, 92% acknowledged the high prestige and income attached to neurosurgery. However, 87% of respondents believed that neurosurgery could impede family life [3]. A strong agreement or agreement to many statements: a competitive training program for neurosurgery, neurosurgeons are intelligent, neurosurgery is an interesting specialty, long operating hours for neurosurgery, neurosurgical illness is challenging and interesting, and whether there should be a compulsory neurosurgery rotation during MBBS were elicited in our survey. Similar to the study in Oman, for instance, these factors influenced the perception of medical students toward neurosurgery. Both students and interns reported that the major obstacle to neurosurgery is the challenging nature of the pathologies in neurosurgery, followed by absence of the neurosurgery residency program, long training periods of neurosurgery specialty, and insufficient exposure during medical school [11]. Similarly, a previous study reported that more than 95% of respondents agreed that neurosurgery necessitates long operation hours as well longer duration of training. Moreover, 93% of the respondents thought that neurosurgery training in Saudi Arabia is too extensive. The acknowledgment that neurosurgery is associated with high prestige and income is a commonly shared perception among medical students where it is looked at as pay back for the challenging specialty and the impedance of neurosurgery on other aspects of life [9].

Regarding the perception of medical students and interns, our study revealed a significant difference between medical students and intern respondents concerning the following statements: a career in neurosurgery, completing undergraduate neurosurgery rotation for MBBS students, completing emergency department rotation for interns, requiring excellent manual dexterity for neurosurgeons, being neurosurgery as an interesting specialty, a compulsory neurosurgery rotation for internship and taking an elective rotation in neurosurgery specialty. Around 40% of medical students agreed or strongly agreed as compared to around 27% of interns when asked if they would consider a career in neurosurgery. Similarly, most Omani medical students and interns considered neurosurgery as a future career with a different degree of interest (interesting in neurosurgery: 71.7%; not interested in selecting neurosurgery: 28.3%) as previously cited [11]. Moreover, 78% of students in Ireland consider neurosurgery as a future career [3]. Most students (86%) were not considering neurosurgery as a future career due to its impact on family life [27]. It is also worth pondering that a large proportion of students might be turning away from a career in neurosurgery because of general anxiety about surgery [1].

In the present study, medical students agreed or strongly agreed more than interns with significant differences in the following statements: intelligent neurosurgeons (medical students: 74.1%, interns: 68%), requiring excellent manual dexterity for neurosurgeons (medical students: 83.7%, interns: 78.6%), neurosurgery as an interesting specialty (medical students: 78.1%, interns: 75.8%), impeding family life (medical students: 60.5%; interns: 63%) and the neurosurgery rotation being compulsory in the internship (medical students: 45.1%, interns: 25.8%). In general, interns had a more negative attitude toward neurosurgery as compared to the medical students. Moreover, around 65% of both students and interns agreed or strongly agreed with the statement that seniors/teachers have a great influence on the student’s choice of specialization after MBBS. In another study in Oman, the existence of a mentor in neurosurgery can raise medical students’ interest in neurosurgery by 98%, followed by the addition of a neurosurgery rotation, and neurosurgery has a high level of reputation and income [11]. However, our study agreed with previous findings [1], where it was reported that a large proportion of students and junior doctors strongly agreed that neurosurgery is an interesting specialty as well as that it requires a high degree of intelligence. Nearly similar findings were also reported in another study [11], where they cited that adding neurosurgery rotation through medical school will have a positive impact and increase the awareness of the neurological conditions for a large percentage of medical students and interns. Concerning the relationship between neurosurgery and family life, our study agreed with the finding of another study from Saudi Arabia [9], where 97.8% of participants in Saudi Arabia believed that neurosurgery could impede family life. Similarly, in Ireland, 70–100% of respondents found neurosurgery can impede family life [3].

Within the career itself, a challenge exists with regard to academic/research vs. clinical vs. mixed of all are diffidently impacting the choice of ultimate career [28]. In our study, almost 67% of the respondents saw themselves going into a purely clinical role, while 17% of the respondents had reportedly envisioned clinician–academician roles for themselves, and 3.60% of respondents had envisioned an academician role only for themselves.

Work burnout is commonly discussed in neurosurgery, and it is thought to be an important influencer on the perception of neurosurgery. The existing study did not have direct questions on this matter; however, the agreement that was seen between both genders and students vs. interns may reflect that influence when participants answered the statement that neurosurgery can impede family life, despite those participants disagreeing with this statement. As mentioned above, SCFHS is the regularity body of residents’ affairs while in training. Over the last decades, SCFHS has implemented many roles on working hours, controlling the relationship between mentors and trainees, etc.

Neurosurgery was always looked at as one of the top medical professions leading to burnout [29] internationally; although there are no burnout studies for neurosurgery staff in Saudi Arabia, multiple studies Saudi studies were conducted for plastic residents [30], orthopedic residents [31], and neurology residents and consultants [32], where all these studies have concluded that moderate to high rates of burnout among the study groups are shown. The fact that neurosurgeons are sharing surgical nature with other surgical specialties, and sharing complexities of pathologies like neurologists, can give a hint of potential high rates of burnout among neurosurgery residents and consultants in Saudi Arabia; however, further specific study for neurosurgery residents and consultants is needed.

This study tried to measure the attitudes and perceptions regarding neurosurgery in a detailed manner, including the factors such as the influence of teachers and role models as well as their future career pathways, etc., which could have a role in their specialty selection. We also tried to get responses from all medical colleges across the Kingdom and were able to get more than a thousand responses. However, one of the major limitations of this study is that it is performed on a convenience sample, which might result in bias as those students who have some strong opinion (positive or negative) about neurosurgery as a specialty might have been more likely to respond to the questionnaire. Another limitation affecting the sample representativeness could be that there was a proportionately higher number of responses from our own institution, i.e., College of Medicine, King Khalid University (though no response was sought from the institution where the participants were enrolled in). Nevertheless, we hope that this study is a useful addition to the literature to understand undergraduate medical students’ attitudes and choices related to neurosurgery as well as specialty selection in general, which can help policy makers and educationalists to develop interventions to promote neurosurgery amongst medical students.

## 5. Conclusions

There was a generally positive perception of neurosurgery as a specialization among medical students, which was greater amongst females. It was also found that there was a great degree of difference in exposure to neurosurgery specialization in the undergraduate program across medical colleges in the Kingdom of Saudi Arabia. Making provisions for exposure of medical students to the functioning of neurosurgery units as well as outreach programs to mentor medical students into a neurosurgery specialization may help in attracting the best talents toward neurosurgery and bridge the gap in the availability of neurosurgery specialists in Saudi Arabia.

## Figures and Tables

**Table 1:** Pro Forma: perceptions and attitudes of medical students and interns toward neurosurgery.

Demographic Factors					
1-Gender:	Male:		Female:		
2-Nationality:	Saudi		Non-Saudi		
3-Year of study:	Year 4	Year 5	Year 6		Interns
4-If you are an intern, have you completed your emergency department rotation?	Yes:		No:		
5-Would you be interested in taking an elective rotation in neurosurgery to get more exposure to the neurosurgery specialty?	Yes:		No:		
Have you been involved directly in management of acute neurosurgical patients?	Yes:		No:		
Have you been involved directly in management of elective neurosurgical patients?	Yes:		No:		
Have you been in operation room for acute neurosurgical procedure?	Yes:		No:		
Have you been in operation room for elective neurosurgical procedure?	Yes:		No:		
6-What kind of role do you see for yourself in your future career?	Academician	Clinician	Administrative		
7-As per your observation, what proportion of your classmates/college friends whom you know well have made up their minds regarding their specialization choices?	Around half	Not sure	Most of them	Few of them	Less than half
Past experience and interest in specialty, Stereotypes and personal factors	Strongly agree	Agree	Disagree	Strongly disagree	Not sure
1-Neurosurgery is an important subject					
2-Neurosurgery is/was taught as part of my undergraduate medical degree					
3-Neurosurgery should be taught as part of an undergraduate medical degree					
4-Neurosurgery rotation should be a compulsory rotation in internship					
5-Neurosurgery is an interesting specialty					
6-Neurosurgery is a dying specialty					
7-Neurosurgeons are well paid					
8-Neurosurgeons are intelligent					
9-Neurosurgeons need to be emotionally detached from their patients					
10-Neurosurgery is a depressing specialty					
11-Neurosurgical treatment does not cure the patients most of the times					
12-Neurosurgery training programs are the most competitive to get into					
13-Neurosurgery is a male specialty					
14-Neurosurgeons need excellent manual dexterity					
15-Neurosurgery is more about technical skill than academic knowledge					
16-I would consider a career in neurosurgery					
17-Neurosurgical illnesses are challenging and interesting					
18-Neurosurgery requires a long training period					
19-Neurosurgery requires long operating hours					
20-Huge prestige is attached to neurosurgery					
21-Work as a neurosurgery specialist can impede family life					
22-Future neurosurgical specialty job opportunities in Saudi Arabia are limited					
23-Adventure loving persons are best suited to do neurosurgery specialization					
24-College teachers/seniors have a great influence on any students’ specialization choice after MBBS					

**Table 2:** Comparison of the item responses between the male and female respondents.

Comparisons between Gender and Items					
Items	Gender	N	Mean	Std. Deviation	*p*-Value
I would consider a career in neurosurgery	Male	518	2.85	1.391	0.374
	Female	496	2.77	1.380	
Neurosurgery training programs are the most competitive to get into	Male	518	3.40	1.143	0.004
	Female	496	3.60	1.050	
Adventure-loving persons are best suited to do neurosurgery specialization	Male	518	3.30	0.996	0.647
	Female	496	3.33	0.905	
College teachers/seniors have a great influence on any students’ specialization choice after MBBS	Male	518	3.73	1.036	0.758
	Female	496	3.75	1.054	
If you are an MBBS student, have you completed your undergraduate (i.e., MBBS program) neurosurgery rotation?	Male	393	1.70	0.457	0.852
	Female	385	1.70	0.459	
Future neurosurgical specialty job opportunities in Saudi Arabia are limited	Male	518	3.26	1.025	0.290
	Female	496	3.32	0.952	
Huge prestige is attached to neurosurgery	Male	518	3.99	0.920	0.894
	Female	496	4.00	0.888	
If you are an intern, have you completed your emergency department rotation?	Male	307	1.78	0.416	0.003
	Female	288	1.67	0.471	
Neurosurgeons are intelligent	Male	518	3.79	0.918	0.000
	Female	496	4.09	0.880	
Neurosurgeons are well paid	Male	518	3.69	1.019	0.670
	Female	496	3.72	0.961	
Neurosurgeons need excellent manual dexterity	Male	518	4.10	0.900	0.124
	Female	496	4.18	0.855	
Neurosurgeons need to be emotionally detached from their patients	Male	518	3.11	1.210	0.274
	Female	496	3.03	1.166	
Neurosurgery is a depressing specialty	Male	518	3.34	1.118	0.003
	Female	496	3.13	1.070	
Neurosurgery is a dying specialty	Male	518	2.97	1.194	0.084
	Female	496	2.89	1.105	
Neurosurgery is a male specialty	Male	518	2.95	1.291	0.000
	Female	496	1.89	1.122	
Neurosurgery is an important subject	Male	518	4.51	0.748	0.030
	Female	496	4.60	0.668	
Neurosurgery is an interesting specialty	Male	518	3.84	1.112	0.000
	Female	496	4.10	0.986	
Neurosurgery is more about technical skill than academic knowledge	Male	518	3.16	1.230	0.663
	Female	496	3.13	1.189	
Neurosurgery is/was taught as part of my undergraduate medical degree	Male	518	3.62	1.093	0.301
	Female	496	3.69	1.061	
Neurosurgery requires a long training period	Male	518	4.28	0.756	0.103
	Female	496	4.36	0.750	
Neurosurgery requires long operating hours	Male	518	4.23	0.747	0.019
	Female	496	4.33	0.725	
Neurosurgery rotation should be a compulsory rotation in internship	Male	518	2.91	1.319	0.014
	Female	496	3.11	1.266	
Neurosurgery should be taught as part of an undergraduate medical degree	Male	518	3.84	1.092	0.296
	Female	496	3.91	1.017	
Neurosurgical illnesses are challenging and interesting	Male	518	3.96	0.908	0.000
	Female	496	4.18	0.796	
Neurosurgical treatment does not cure the patients most of the times	Male	518	3.16	1.131	0.159
	Female	496	3.06	1.026	
Working as a neurosurgery specialist can impede family life	Male	518	3.87	0.877	0.000
	Female	496	3.65	0.871	
Would you be interested in taking an elective rotation in neurosurgery to get more exposure to the neurosurgery specialty?	Male	518	1.59	0.493	0.020
	Female	496	1.52	0.500	

**Table 3:** Comparison of item responses between medical students and interns.

Comparisons between Items and Students Groups (Interns and Medical Students)					
Items	Year of Study	Frequency	Mean	Std. Deviation	*p*-Values
I would consider a career in neurosurgery	Students	630	2.99	1.379	0.000
	Interns	384	2.51	1.344	
Neurosurgery training programs are the most competitive to get into	Students	630	3.53	1.094	0.247
	Interns	384	3.44	1.116	
Adventure-loving persons are best suited to do neurosurgery specialization	Students	630	3.35	0.950	0.076
	Interns	384	3.24	0.952	
College teachers/seniors have a great influence on any students’ specialization choice after MBBS	Students	630	3.77	1.004	0.149
	Interns	384	3.68	1.105	
If you are an MBBS student, have you completed your undergraduate (i.e., MBBS program) neurosurgery rotation?	Students	547	1.78	0.418	0.000
	Interns	231	1.53	0.500	
Future neurosurgical specialty job opportunities in Saudi Arabia are limited	Students	630	3.24	0.967	0.049
	Interns	384	3.36	1.023	
Huge prestige is attached to neurosurgery	Students	630	3.99	0.900	0.945
	Interns	384	3.99	0.911	
If you are an intern have you completed your emergency department rotation?	Students	220	1.87	0.334	0.000
	Interns	375	1.64	0.481	
Neurosurgeons are intelligent	Students	630	4.01	0.865	0.001
	Interns	384	3.81	0.971	
Neurosurgeons are well paid	Students	630	3.71	1.000	0.618
	Interns	384	3.68	0.977	
Neurosurgeons need excellent manual dexterity	Students	630	4.19	0.853	0.027
	Interns	384	4.06	0.916	
Neurosurgeons need to be emotionally detached from their patients	Students	630	3.03	1.168	0.151
	Interns	384	3.14	1.220	
Neurosurgery is a depressing specialty	Students	630	3.20	1.096	0.139
	Interns	384	3.30	1.102	
Neurosurgery is a dying specialty	Students	630	2.93	1.149	0.916
	Interns	384	2.93	1.158	
Neurosurgery is a male specialty	Students	630	2.40	1.350	0.301
	Interns	384	2.49	1.270	
Neurosurgery is an important subject	Students	630	4.57	0.700	0.221
	Interns	384	4.52	0.730	
Neurosurgery is an interesting specialty	Students	630	4.04	1.047	0.005
	Interns	384	3.85	1.071	
Neurosurgery is more about technical skill than academic knowledge	Students	630	3.17	1.202	0.452
	Interns	384	3.11	1.224	
Neurosurgery is/was taught as part of my undergraduate medical degree	Students	630	3.61	1.094	0.100
	Interns	384	3.73	1.047	
Neurosurgery requires a long training period	Students	630	4.35	0.740	0.158
	Interns	384	4.28	0.774	
Neurosurgery requires long operating hours	Students	630	4.26	0.739	0.300
	Interns	384	4.31	0.737	
Neurosurgery rotation should be a compulsory rotation in internship	Students	630	3.25	1.266	0.000
	Interns	384	2.61	1.249	
Neurosurgery should be taught as part of an undergraduate medical degree	Students	630	3.91	1.059	0.182
	Interns	384	3.82	1.050	
Neurosurgical illnesses are challenging and interesting	Students	630	4.10	0.835	0.119
	Interns	384	4.01	0.901	
Neurosurgical treatment does not cure the patients most of the times	Students	630	3.12	1.037	0.774
	Interns	384	3.10	1.152	
Working as a neurosurgery specialist can impede family life	Students	630	3.76	0.863	0.754
	Interns	384	3.77	0.910	
Would you be interested in taking an elective rotation in neurosurgery to get more exposure to the neurosurgery specialty?	Students	630	1.47	0.499	0.000
	Interns	384	1.70	0.460	

**Table 4:** Gender-wise comparison (male and female) views with items (strongly agree and agree combined).

Gender Wise Comparison (Male and Female) Views with Items (Strongly Agree and Agree Combined)	
**I Would Consider a Career in Neurosurgery**	
Male	36.9%
Female	33.1%
**Neurosurgery training programs are the most competitive to get into**	
Male	49.4%
Female	55.5%
***Adventure-loving persons are best suited to do neurosurgery specialization***	
Male	40.5%
Female	37.9%
***College teachers/seniors have a great influence on any students’ specialization choice after MBBS***	
Male	64.9%
Female	65.3%
***Huge prestige is attached to neurosurgery***	
Male	70.5%
Female	72.4%
***Neurosurgeons are intelligent***	
Male	67.4%
Female	76.6%
***Neurosurgeons are well paid***	
Male	55.6%
Female	55.2%
***Neurosurgeons need excellent manual dexterity***	
Male	79.7%
Female	83.9%
***Neurosurgeons need to be emotionally detached from their patients***	
Male	40.0%
Female	36.3%
***Neurosurgery is a depressing specialty***	
Male	43.6%
Female	37.7%
***Neurosurgery is a dying specialty***	
Male	32.2%
Female	28.4%
***Neurosurgery is a male specialty***	
Male	36.5%
Female	12.5%
***Neurosurgery is an important subject***	
Male	94.2%
Female	96.0%
***Neurosurgery is an interesting specialty***	
Male	72.0%
Female	82.7%
***Neurosurgery is more about technical skill than academic knowledge***	
Male	44.4%
Female	40.7%
***Neurosurgery is/was taught as part of my undergraduate medical degree***	
Male	59.3%
Female	59.1%
***Neurosurgery requires a long training period***	
Male	87.6%
Female	89.1%
***Neurosurgery requires long operating hours***	
Male	84.7%
Female	88.1%
***Neurosurgery rotation should be a compulsory rotation in internship***	
Male	35.5%
Female	40.1%
***Neurosurgery should be taught as part of an undergraduate medical degree***	
Male	70.7%
Female	70.6%
***Neurosurgical illnesses are challenging and interesting***	
Male	79.2%
Female	87.1%
***Neurosurgical treatment does not cure the patients most of the times***	
Male	39.2%
Female	34.9%
***Working as a neurosurgery specialist can impede family life***	
Male	65.3%
Female	57.5%

## Data Availability

All data generated or analyzed during this study are included in this article. Further enquiries can be directed to the corresponding author.

## Supplementary Materials

The following supporting information can be downloaded at: https://www.mdpi.com/article/10.3390/medicina58081120/s1, Table S1—Comparisons of students (Interns and students) views with items (strongly agree and agree combined), Table S2—Comparisons between interns who have neurosurgery exposure vs. interns who did not have exposure to neurosurgery, Table S3—Comparisons between Students who have neurosurgery exposure vs. students who did not have exposure to neurosurgery; Figure S1—Diagram shows the 7-year-pathway of medical students in Saudi Arabia after graduation from high school.
