# Supplementary material for: Perceptions of Neurosurgery among Medical Students and Interns: A National Cross-Sectional Study
Source: Medicina (Kaunas). 2022 Aug 18;58(8):1120. doi: 10.3390/medicina58081120 (PMC9412531; doi:10.3390/medicina58081120)
Supplement: Supplementary file 1 [file medicina-58-01120-s001.zip › medicina-1836158-supplementary.pdf]

**Table S1. Comparisons of students (Interns and students) views with items (strongly agree and agree combined)**

| Comparisons of students (Interns and medical students) views with items (strongly agree and agree combined) | |
|---|---|
| *I would consider a career in neurosurgery* | |
| Medical Students | 40.2% |
| Interns | 26.6% |
| *Neurosurgery training programs are the most competitive to get into* | |
| Medical Students | 52.9% |
| Interns | 51.0% |
| *Adventure loving persons are best suited to do neurosurgery specialization* | |
| Medical Students | 41.1% |
| Interns | 36.2% |
| *College teachers/seniors have a great influence on any students' specialization choice after MBBS.* | |
| Medical Students | 65.4% |
| Interns | 65.0% |
| *Future neurosurgical specialty job opportunities in Saudi Arabia are limited* | |
| Medical Students | 34.4% |
| Interns | 40.9% |
| *Huge prestige is attached to neurosurgery* | |
| Medical Students | 71.1% |
| Interns | 71.9% |
| *Neurosurgeons are intelligent* | |
| Medical Students | 74.1% |
| Interns | 68.0% |
| *Neurosurgeons are well paid* | |
| Medical Students | 54.9% |
| Interns | 56.3% |
| *Neurosurgeons need excellent manual dexterity* | |
| Medical Students | 83.7% |
| Interns | 78.6% |
| *Neurosurgeons need to be emotionally detached from their patients* | |
| Medical Students | 35.2% |
| Interns | 43.0% |
| **Neurosurgery is a depressing specialty** | |
| Medical Students | 38.7% |
| Interns | 44.0% |
| *Neurosurgery is a dying specialty* | |

| | |
|---|---|
| Medical Students | 29.4% |
| Interns | 32.0% |
| **Neurosurgery is a male specialty** | |
| Medical Students | 24.4% |
| Interns | 25.3% |
| ***Neurosurgery is an important subject*** | |
| Medical Students | 95.6% |
| Interns | 94.3% |
| **Neurosurgery is an interesting specialty** | |
| Medical Students | 78.1% |
| Interns | 75.8% |
| ***Neurosurgery is more about technical skill than academic knowledge*** | |
| Medical Students | 43.7% |
| Interns | 40.9% |
| ***Neurosurgery is/was taught as part of my undergraduate medical degree*** | |
| Medical Students | 55.2% |
| Interns | 65.6% |
| **Neurosurgery requires a long training period** | |
| Medical Students | 89.0% |
| Interns | 87.2% |
| ***Neurosurgery requires long operating hours*** | |
| Medical Students | 85.1% |
| Interns | 88.5% |
| ***Neurosurgery rotation should be a compulsory rotation in internship*** | |
| Medical Students | 45.1% |
| Interns | 25.8% |
| ***Neurosurgery should be taught as part of an undergraduate medical degree*** | |
| Medical Students | 70.3% |
| Interns | 71.1% |
| ***Neurosurgical illnesses are challenging and interesting*** | |
| Medical Students | 84.1% |
| Interns | 81.3% |
| ***Neurosurgical treatment does not cure the patients most of the times*** | |
| Medical Students | 34.8% |
| Interns | 40.9% |
| ***Work as a neurosurgery specialist can impede family life*** | |
| Medical Students | 60.5% |
| Interns | 63.0% |

**Table S2. Comparisons between interns who have neurosurgery exposure vs. interns who did not have exposure to neurosurgery.**

| Item | Yes (have exposure) | | | Have no exposure | | | p-value |
|---|---|---|---|---|---|---|---|
| | Mean | N | Std. Deviation | Mean | N | Std. Deviation | |
| Adventure loving persons are best suited to do neurosurgery specialization | 3.4 | 163 | 1.0 | 3.3 | 432 | .9 | 0.16 |
| College teachers/seniors have a great influence on any students' specialization choice after MBBS. | 3.7 | 163 | 1.1 | 3.7 | 432 | 1.0 | 0.98 |
| Future neurosurgical specialty job opportunities in Saudi Arabia are limited | 3.3 | 163 | 1.1 | 3.4 | 432 | .9 | 0.73 |
| Huge prestige is attached to neurosurgery | 3.9 | 163 | .9 | 4.0 | 432 | .9 | 0.28 |
| I would consider a career in neurosurgery | 2.9 | 163 | 1.3 | 2.7 | 432 | 1.3 | 0.11 |
| Neurosurgeons are intelligent | 3.9 | 163 | 1.0 | 3.9 | 432 | .9 | 0.99 |
| Neurosurgeons are well paid | 3.7 | 163 | 1.0 | 3.7 | 432 | 1.0 | 0.83 |
| Neurosurgeons need excellent manual dexterity | 4.0 | 163 | .9 | 4.1 | 432 | .9 | 0.10 |
| Neurosurgeons need to be emotionally detached from their patients | 3.3 | 163 | 1.2 | 3.2 | 432 | 1.1 | 0.18 |
| Neurosurgery is a depressing specialty | 3.5 | 163 | 1.1 | 3.3 | 432 | 1.1 | 0.08 |
| Neurosurgery is a dying specialty | 2.9 | 163 | 1.2 | 3.0 | 432 | 1.1 | 0.29 |

| | | | | | | | |
|---|---|---|---|---|---|---|---|
| Neurosurgery is a male specialty | 2.5 | 163 | 1.3 | 2.5 | 432 | 1.3 | 0.76 |
| Neurosurgery is an important subject | 4.4 | 163 | .9 | 4.6 | 432 | .6 | **0.01** |
| Neurosurgery is an interesting specialty | 3.9 | 163 | 1.0 | 3.9 | 432 | 1.1 | 0.47 |
| Neurosurgery is more about technical skill than academic knowledge | 3.2 | 163 | 1.3 | 3.2 | 432 | 1.2 | 0.91 |
| Neurosurgery is/was taught as part of my undergraduate medical degree | 3.9 | 163 | .9 | 3.7 | 432 | 1.1 | **0.01** |
| Neurosurgery requires a long training period | 4.2 | 163 | .8 | 4.3 | 432 | .7 | 0.39 |
| Neurosurgery requires long operating hours | 4.3 | 163 | .8 | 4.3 | 432 | .7 | 0.61 |
| Neurosurgery rotation should be a compulsory rotation in internship: | 2.9 | 163 | 1.2 | 2.9 | 432 | 1.3 | 0.93 |
| Neurosurgery should be taught as part of an undergraduate medical degree | 4.0 | 163 | .9 | 3.8 | 432 | 1.1 | 0.10 |
| Neurosurgery training programs are the most competitive to get into. | 3.5 | 163 | 1.1 | 3.5 | 432 | 1.1 | 0.75 |
| Neurosurgical illnesses are challenging and interesting | 4.0 | 163 | .9 | 4.0 | 432 | .9 | 0.57 |
| Neurosurgical treatment does not cure the patients most of the times | 3.2 | 163 | 1.1 | 3.2 | 432 | 1.1 | 0.80 |
| Work as a neurosurgery specialist can impede family life | 3.8 | 163 | .9 | 3.8 | 432 | .8 | 0.86 |

| | | | | | | | |
|---|---|---|---|---|---|---|---|
| Would you be interested in taking an elective rotation in neurosurgery to get more exposure of the neurosurgery specialty? | 1.6 | 163 | .5 | 1.6 | 432 | .5 | 0.42 |

**Table S3. Comparisons between Students who have neurosurgery exposure vs. students who did not have exposure to neurosurgery.**

| Item | Yes (Have Exposure) | | | No (No Exposure) | | | p-value |
|---|---|---|---|---|---|---|---|
| | Mean | Frequency | SD | Mean | Frequency | Std. Deviation | |
| Adventure loving persons are best suited to do neurosurgery specialization | 3.237 | 232 | 1.006 | 3.361 | 546 | 0.940 | 0.101 |
| College teachers/seniors have a great influence on any students' specialization choice after MBBS. | 3.871 | 232 | 1.011 | 3.683 | 546 | 1.080 | **0.024** |
| Future neurosurgical specialty job opportunities in Saudi Arabia are limited | 3.233 | 232 | 1.064 | 3.342 | 546 | 0.978 | 0.164 |
| Huge prestige is attached to neurosurgery | 3.970 | 232 | 0.918 | 4.051 | 546 | 0.899 | 0.251 |
| I would consider a career in neurosurgery | 2.677 | 232 | 1.452 | 2.952 | 546 | 1.374 | **0.012** |
| Neurosurgeons are intelligent | 3.819 | 232 | 0.977 | 4.029 | 546 | 0.875 | **0.003** |
| Neurosurgeons are well paid | 3.728 | 232 | 1.032 | 3.736 | 546 | 1.017 | 0.922 |
| Neurosurgeons need excellent manual dexterity | 4.017 | 232 | 0.949 | 4.192 | 546 | 0.870 | **0.013** |

| | | | | | | | |
|---|---|---|---|---|---|---|---|
| Neurosurgeons need to be emotionally detached from their patients | 3.086 | 232 | 1.217 | 3.097 | 546 | 1.199 | 0.908 |
| Neurosurgery is a depressing specialty | 3.250 | 232 | 1.104 | 3.223 | 546 | 1.127 | 0.762 |
| Neurosurgery is a dying specialty | 2.871 | 232 | 1.136 | 2.982 | 546 | 1.175 | 0.224 |
| Neurosurgery is a male specialty | 2.582 | 232 | 1.362 | 2.385 | 546 | 1.343 | 0.062 |
| Neurosurgery is an important subject | 4.552 | 232 | 0.662 | 4.553 | 546 | 0.718 | 0.982 |
| Neurosurgery is an interesting specialty | 3.892 | 232 | 1.053 | 4.068 | 546 | 1.057 | 0.034 |
| Neurosurgery is more about technical skill than academic knowledge | 3.177 | 232 | 1.173 | 3.158 | 546 | 1.233 | 0.840 |
| Neurosurgery requires a long training period | 4.284 | 232 | 0.759 | 4.348 | 546 | 0.752 | 0.283 |
| Neurosurgery requires long operating hours | 4.224 | 232 | 0.745 | 4.324 | 546 | 0.729 | 0.082 |
| Neurosurgery rotation should be a compulsory rotation in internship: | 2.978 | 232 | 1.327 | 3.172 | 546 | 1.278 | 0.056 |
| Neurosurgery should be taught as part of an undergraduate medical degree | 4.086 | 232 | 0.940 | 3.842 | 546 | 1.091 | **0.003** |
| Neurosurgery training programs are the most competitive to get into. | 3.366 | 232 | 1.124 | 3.604 | 546 | 1.094 | **0.006** |
| Neurosurgical illnesses are | 4.099 | 232 | 0.880 | 4.093 | 546 | 0.882 | 0.934 |

| | | | | | | | |
|---|---|---|---|---|---|---|---|
| challenging and interesting | | | | | | | |
| Neurosurgical treatment does not cure the patients most of the times | 2.974 | 232 | 1.116 | 3.176 | 546 | 1.071 | 0.018 |
| Work as a neurosurgery specialist can impede family life | 3.849 | 232 | 0.862 | 3.777 | 546 | 0.893 | 0.295 |
| Would you be interested in taking an elective rotation in neurosurgery to get more exposure of the neurosurgery specialty? | 1.578 | 232 | 0.495 | 1.498 | 546 | 0.500 | **0.043** |

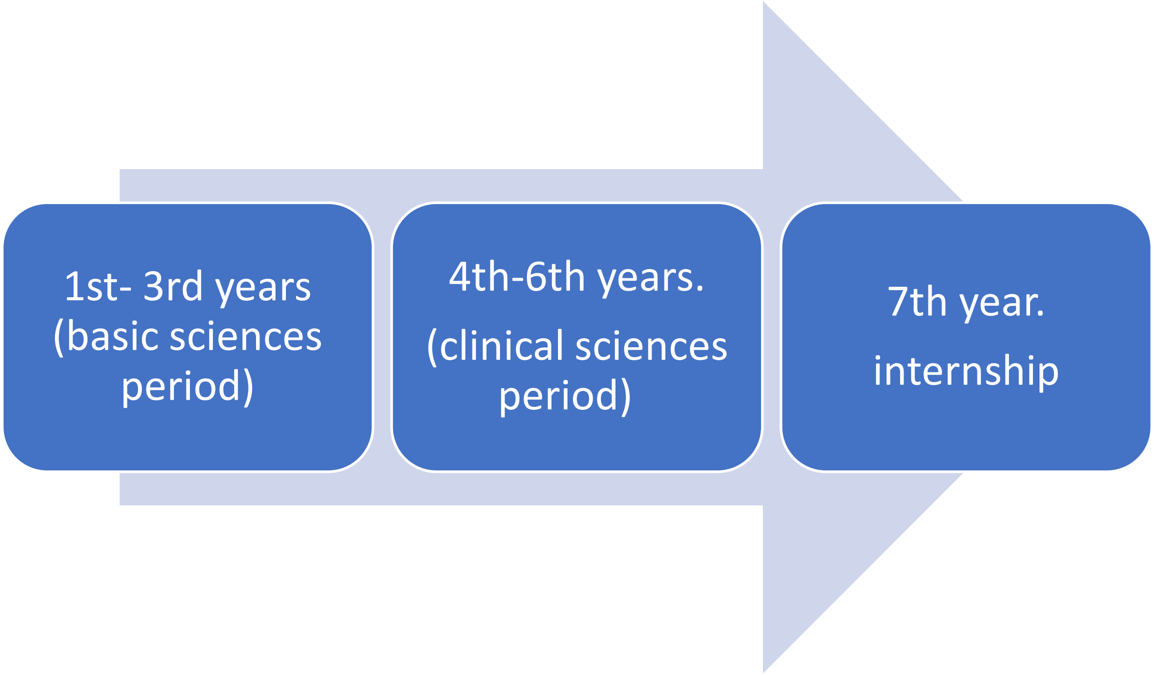


**Figure S1. diagram shows the 7-year-pathway of medical students in Saudi Arabia after graduation from high school.**
